# Molecular Characterization and Diversity of Bacteria Isolated from Fish and Fish Products Retailed in Kenyan Markets

**DOI:** 10.1155/2022/2379323

**Published:** 2022-07-18

**Authors:** Domitila Ndinda Kyule, John M. Maingi, Ezekiel Mugendi Njeru, Anthony Kebira Nyamache

**Affiliations:** ^1^Department of Biochemistry, Microbiology and Biotechnology, Kenyatta University, 43844-00100 Nairobi, Kenya; ^2^Kenya Marine and Fisheries Research Institute (KMFRI), 451-10230 Sagana, Kenya

## Abstract

Fish products are highly vulnerable to microbial contamination due to their soft tissues, making them perishable and harmful to consumers. The clinical and subclinical infections reported by fish consumers are mainly associated with pathogenic microorganisms in fish products. Therefore, this study aimed at establishing the molecular profiles and diversity of the bacterial isolates from fish and fish products obtained from Kirinyaga County markets in Kenya. A total of 660 samples were randomly sampled in six Kirinyaga County markets and transported to Kenyatta University for bacterial isolation. The fish skin surface was cut using a sterile knife and blended in buffered peptone water. The blended product was serially diluted and plated on nutrient agar. After 24 hours, the bacteria cultures were subcultured to obtain pure bacterial isolates. The pure isolates were grouped and characterized based on their morphology and biochemical characteristics. One representative of each group was selected for bacterial DNA extraction. The 16S rRNA gene was amplified using the 27F and 1492R primers, and the obtained PCR product was subjected to Sanger-based sequencing using the same primers. Morphological characterization yielded 54 morpho groups. Phylogenetic analysis revealed diverse bacterial strains, including *Escherichia coli*, *Salmonella enterica*, *Citrobacter freundii*, *Bacillus* sp. and *Alcaligenes faecalis. Bacillus* sp. was the most dominant group, as compared to other isolates in the study. The study, therefore, revealed diverse bacterial strains from the fish products. This high microbial diversity calls for heightened surveillance to prevent possible foodborne disease outbreaks.

## 1. Introduction

Traditionally, fish has formed a large part of the human diet, and it is the main supply of animal protein in many parts of the world [1]. According to Thilsted et al. [2], fish meat has a high nutrient profile and is more nuanced than animal meat. Fish and fish products are tremendously digestible with high levels of omega-3 polyunsaturated fatty acids (PUFAs) and vitamins [3]. Fish also plays a protective role, especially in controlling high blood pressure, obesity, coronary heart disease, stroke, and others [4]. In most developing countries, fish act as the sole source of affordable nutrition, causing a rise in demand which is expected to rise by up to 7 kg per capita in early 2030 [5]. According to FAO [5], global fish production increased dramatically by 2018 hitting 179 million tons. However, the safety of fish products has been highly compromised due to contamination by pathogenic microbes leading to spoilage and outbreak of foodborne diseases [1].

Fish products are highly susceptible to microbial contamination, which creates a health hazard. Contaminated fish products have been associated with the transmission of many established foodborne microbial infections and intoxications [4, 6]. The contamination may originate from uncleaned hands and surfaces during cleaning and evisceration of fish [7]. Spoilage and pathogenic microbes can be introduced into fish products at any point throughout the supply chain. According to Böhme et al. [8], freezing alone does not prevent microbial contamination of fish because of autolytic activities and chemical changes occur in fish after harvest. Fish spoilage is accelerated by microorganisms associated with aquatic environments as well as contaminants during postharvest handling.

Pathogenic bacteria associated with fish and fish products are categorized into three general groups. The first group is the indigenous bacteria which belong to the natural micro-flora of fish. These include *Clostridium botulinum*, pathogenic *Vibrio* sp., and *Aeromonas hydrophila* [8]. The second group is the enteric bacteria, commonly known as non indigenous bacteria. This group of bacteria is present in fish and fish products due to fecal contamination. *Salmonella* sp., *Shigella* sp., pathogenic *Escherichia coli*, *and Staphylococcus aureus* are common examples [9]. The third group is bacterial isolates from fish and fish products and is considered as potential contaminants that can occur during processing, storage, or cooking. These bacteria include *Bacillus cereus*, *Listeria monocytogenes*, *Staphylococcus aureus*, *Clostridium perfringens*, and *Salmonella* sp. [10]. In addition, improper storage and handling of fishery products may lead to increased growth of spoilage bacteria such as *Lactobacillus* sp., *Proteus* sp., *Shewanella putrefaciens*, and *Pseudomonas* sp. [11].

In Africa, there is a rapid expansion of fish markets due to intensive culture by smallholder farmers. However, various bacterial contaminants have negatively affected fish and its products, contributing to contamination. The fish and fish products spoilage problem have been associated with high temperatures, poor infrastructure, and long market distances. These factors contribute to the proliferation of bacterial contaminants [12]. The current traditional handling and preservation methods of fishery products significantly contribute to the loss of quality. Bulk storage of fish by farmers in the region also leads to a partial or complete deterioration of fish quality. Fish handling during processing determines the quality of the final product. In Kenya, there is an increase in fish consumption, hence the need to understand microbial contamination, which is a great public health concern. This is due to the apparent occurrence and increase of bacterial contaminants in fish products from markets which poses significant health implications to consumers and fish vendors. Currently, there is little or no information regarding profiles of bacterial contaminants that can allow an informed opinion of risks associated with handling and consumption of fish products in the region [13].

This study hypothesized that bacterial isolates from fish and fish products in Kenyan markets have distinct profiles and genetic diversities. The study objectives were to isolate and characterize the bacterial isolates based on their morphological and molecular characteristics and to determine their genetic diversities.

## 2. Materials and Methods

### 2.1. Experimental Site and Sampling Design

The study was carried out in Kirinyaga County, Kenya. The region lies at latitude 0°34′23.43″S and longitude 37°19′31.7″E. The main economic activities in this county are livestock, crop, and fish farming. The inclusion criteria of the selected markets included markets that sell fish and fish products directly to the consumers, sell at least fish and other three fish related-products, and operate daily and in known geographical locations. The cluster sampling design was employed in this study [14], where each market was clustered into five quadrants by use of the main road in the food markets. In each quadrant, proportionate sampling based on the number of fish products vendors in each cluster was done to get the number of participants. Random tables were used to randomly identify the participants, while the convenient sampling strategy described by Maina et al. [15] was used to randomly sample the fish and fish products in the selected fish retail markets in the county. These markets included Kerugoya, Ndia, Kianyaga, Mwea, Sagana, and Tebere.

### 2.2. Sample Collection

A purposive study design was used in this study. A total of 60 samples of each fish product (raw fish, sausages, samosas, skewers, fried fish, soup, balls, cakes, hot dogs, fingers, and burgers) were randomly sampled from fish vendors in the six selected markets in Kirinyaga County. The samples were separately packaged in zip-locked bags and transported in cool boxes to microbiology laboratory at Kenyatta University for analysis. Raw fish were sampled and skinned aseptically. About 10 g of flesh and skin from the fish was used and homogenized with 90 ml of buffered peptone water for 3 minutes using a Kenwood blender model BLP15.150BK, UK (model number, country of origin). Other fish products were prepared by cutting 10 g of each sample into small pieces using a sterile knife. The cut samples were further ground in a Kenwood blender with 90 ml of buffered peptone water. Each fish sample was a composite sample obtained from 3 fish products of the same type.

### 2.3. Isolation of Bacteria and Morphological Characterization

Ten fold serial dilution was prepared in physiological saline (0.85% NaCl). After blending, a mixture of 1 ml was diluted using sterile peptone water at ratios 1 : 10, 1 : 100, 1 : 1000, and 1 : 10000. A 0.1 ml of each dilution was spread on nutrient agar (NA) medium and incubated at 37°C for 24 hours; after which, plates with bacterial colonies ranging from 30 to 300 colony forming units (CFUs) were selected; and the colonies were counted to establish the colony-forming units (CFUs) of each sample. The inoculated plates of each sample were checked for distinct colonies. The distinct colonies were then picked for subsequent subculturing to obtain pure isolates. Purified bacterial isolates were grouped based on their morphological characteristics. All the isolated bacteria were subjected to Gram stain procedure to help group the bacteria into Gram-negative and Gram-positive groups [16]. After that, the isolates were characterized based on microbiological analysis using the following biochemical tests: citrate utilization, triple sugar iron test, urease test, indole test, carbohydrate fermentation, and sulfide indole motility test [17]. The bacterial isolates were grouped based on their similarities in morphology, Gram stain, and biochemical characteristics. After the morphological and biochemical characterization, a representative of each group was selected randomly for molecular characterization.

### 2.4. Molecular Characterization of Bacterial Isolates

#### 2.4.1. DNA Extraction

Purified bacterial isolates were inoculated on nutrient agar and incubated at 37°C 24 hr. before the DNA extraction. The Qiagen bacterial DNA extraction kit (Siegen, Germany) was used to extract the genomic DNA following the manufacturer's protocol. The quality of the extracted DNA was determined by subjecting the samples to gel electrophoresis. Three microliters (3 *μ*l) of each sample were stained using the SYBR green dye and loaded in a 1% agarose gel at 80 V in 0.5X TBE buffer (Bio lab) for 30 minutes.

#### 2.4.2. PCR Amplification

The isolated DNA was amplified based on a protocol developed by Ramanadevi et al. [18]. The 16S rRNA gene was amplified using the primers 27F (5-′AGAGTTTGATCMTGGCTCAG-3′) and 1492R (5′-TACGGYTACCTTGTTA CGACTT-3′). The PCR amplification was done in a total reaction volume of 25 *μ*l PCR master mix containing 1 *μ*l DNA template, 12.5 *μ*l dots, 1× Taw polymerase buffer (Mgcl_2_), 5× buffer 3 *μ*l PCR water, and 0.25 *μ*l of each primer. Amplification conditions were initial denaturation at 94°C for 2 min then at 94°C for 45 s, annealing at 62°C for 45 s, and extension at 72°C for 2 min. This was followed by a final extension at 72°C for 5 min and a final hold at 4°C. The final PCR products were visualized on 1% agarose gel electrophoresis to confirm whether the PCR process was successful. The PCR product was stained with ethidium bromide, loaded onto the gel wells, and visualized using a transilluminator (Biostep, Germany) [19].

#### 2.4.3. Sequencing

The obtained PCR products were purified using the QIAquick PCR purification kit (QIAGEN, Netherlands) following the manufacturer's instructions. These products were then directly sequenced using automated DNA sequencer ABI 377 (Applied Biosystems, Foster City, USA), using fluorescence-labeled dideoxynucleotide chain terminators (ABI Prism BigDye™ Terminator Cycle Sequencing Reaction kit; Applied Biosystems).

#### 2.4.4. Data Analysis

Forward and reverse sequences of the bacterial 16S rRNA gene-based sequence data were analyzed using Finch-Tv software Version 1.4.0. Consensus sequences were generated using CLC Genomics Workbench 10 software. Similar sequences of the consensus were searched using BLAST available in the NCBI site (https://blast.ncbi.nlm.nih.gov/Blast.cgi). The matching organism sequences were obtained from the same database and used as the reference sequences. Multiple sequence alignment were done using the CLUSTAL W. option in the MegaX software. The phylogenetic analysis was done using the neighbor-joining method based on Tamura-Nei with 1000 bootstrap replication and the phylogenetic tree drawn using the MEGA X (version 10.1.8). The resulting consensus sequences were deposited on the NCBI Gene Bank database, and accession numbers were obtained. Furthermore, the nucleotide diversity of the 16S rRNA gene sequences was calculated using DnaSP 6 software. Additionally, genetic diversity and conversion of sequence data to haplotypes were done using DNA SP6 software. The analysis of genetic differentiation was computed using the Arlequin software version 3.5.2.2, and genetic differentiation was determined by computing pairwise F_ST_ matrix (Fixation index) where the distance among haplotypes was used.

## 3. Results

### 3.1. Morphological Characteristics

**Figure 1:**
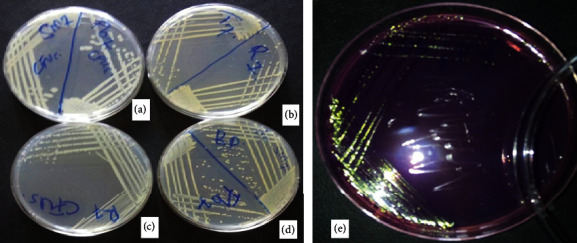
Purified bacterial isolates from fish and fish products on nutrient agar medium. (a) H21-FCTM4, (b) A9-WCTM6, (c) J6-WTTM5, (d) H12-BCTM5, and (e) green metallic sheen characteristic in EMB medium for isolate M10-SMCTM1.

A total of 158 pure bacterial isolates were obtained from the fish and fish products sampled from different markets. Based on the morphological and biochemical characteristics, the isolates were placed into 54 morphological groups. Majority of the isolates (27 isolates) were from raw fish. The isolated bacteria had round colonies of approximately 3 mm and were translucent (Table 1). In addition, other isolates were cream white and had shiny golden yellow colonies ranging from 1 to 1.5 mm, while some had black spots at the center. Besides, some colonies were round and whitish with slightly smooth margin in nutrient agar (NA) (Figure 1). Processed fish products had fewer number of isolates compared to raw fish. The isolates obtained from samosa had five distinct bacterial colonies, ranging from cream to white, while some were translucent with a black spot at the center. Besides, they had round flat colonies with smooth margins. Sausages had afew large bacterial isolates of approximately 4 mm, translucent, and black spot at the center (Figure 1). Based on the Gram staining reaction, 24 of the total isolates were Gram-positive, while the rest were Gram-negative. All the Gram-positive isolates were rod-shaped except three isolates, which were cocci-shaped. Majority (25 isolates) of the isolates were urease negative, while 21 isolates were urease positive since they were able to hydrolyze urea. Some isolates (16) were indole test positive by formation of pink-red suspension, while the others were negative. In addition, 20 isolates were able to utilize glucose under low pH hence methyl-red positive and 26 isolates appeared yellow, hence methyl-red negative (Table 2).

### 3.2. Phylogenetic Analysis

**Figure 2:**
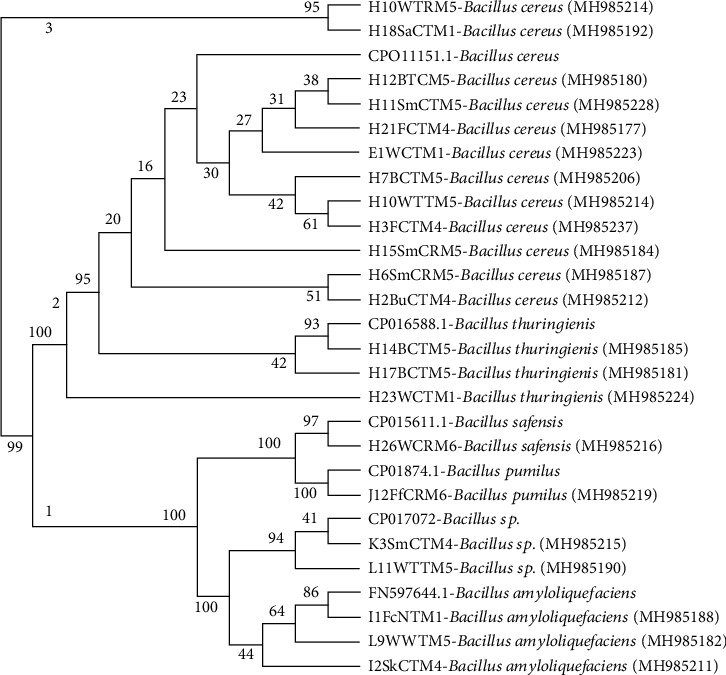
Phylogenetic tree with subclusters A, B, C, and D showing the relationships among the bacterial isolates from raw fish and various fish products and reference isolates. Bootstrap values indicated at each node. Ref: reference strains from NCBI.

Phylogenetic analysis of the 16S ribosomal RNA gene revealed that *Bacillus* sp. were the most predominant bacterial strains isolated from the fish products. The sequences from this group clustered the bacterial isolates into three main clusters (1–3). Cluster 1 comprised bacterial isolates I1FcNTM1, L9WWTM5, and I2SkCTM4 which were closely related to *Bacillus amyloliquefaciens*. In addition, cluster 1 had bacterial isolates K3SmCTM4 and L11WTTM5 closely matched to *Bacillus* sp. Majority of the bacterial isolates clustered in cluster 2 (Figure 2). Bacterial isolates H17BTCM5, H14BCTM5, and H23WCTM1 in cluster 2 were closely related to *Bacillus thuringiensis*, while isolates H12BCTM5, H11SmCTM5, H21FCTM4, E1WCTM1, H7BCTM5, H10WTTM5, H3FCTM4, H15SmCTM5, H6SmCRM5, and H2BuCTM4 were closely related to *Bacillus cereus*. Cluster 3 had only two isolates H10WTRM5 and H18SaCTM1, which had no close reference matches in the NCBI database (Figure 2).

**Figure 3:**
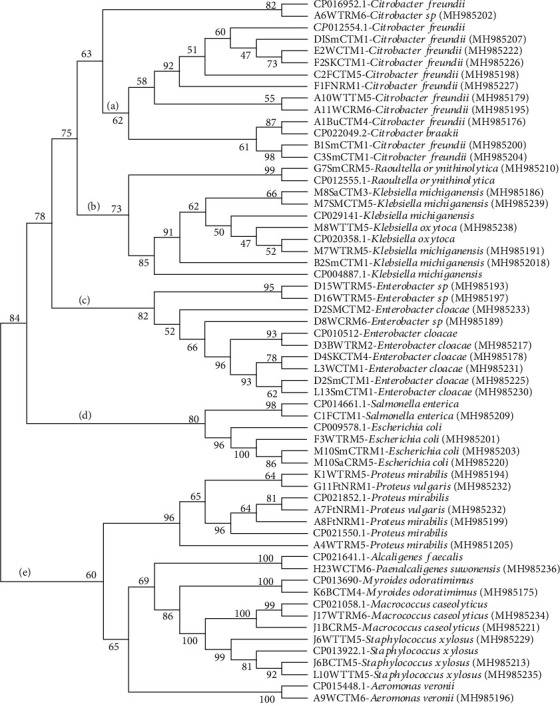
Phylogenetic tree with subclusters A, B, C, and D showing the relationships of bacterial 16srRNA sequences obtained from fish and fish products aligned with reference sequences from the NCBI database. Bootstrap values indicated at each node. Ref: reference strains from NCBI.

The 16S rRNA gene-based sequence also revealed other diverse bacterial isolates (Figure 3). Phylogenetic analysis grouped these isolates into three main clusters (clusters A, B, and C). Cluster A had majority, and it comprised of bacterial isolates, C2FCTM5, E2WCTM1, D1SmCTM1, A11WCRM6, F2SkCTM1, and F1FfNRM1, which were closely related to *Citrobacter freundii*. Bacterial isolates B1SmCTM1 and C3SmCTM1 were closely matched with *Citrobacter braakii*, while isolates B2SmTM1, M8SaCTM3, and M7SmCTM5 were closely related to *Klebsiella michiganensis*. Isolates M8WTTM5 and M7WTRM5 were closely related to *Klebsiella oxytoca*, while bacterial isolate G7SmCRM5 was closely related to *Raoultella omithinolytica*. Bacterial isolates D2SmCTM2, D3BWTRM2, D15WTRM5, D16WTTM5, D8WCRM6, D4SkCTM4, and L3WCTM1 were closely matched to *Enterobacter cloacae* also in cluster A. Cluster B had the lowest number of isolates with bacterial isolate C1FCTM1 closely matched to *Salmonella enterica* and bacterial isolates; F3WTRM5, M10SmCTM1, and M10SaCRM5 closely related to *Escherichia coli* among other isolates (Figure 3). In cluster E, bacterial isolates A8FtNRM1, A7FtNRM1, G11FfNRM1, K1WTRM5, and A4WTRM5 were closely matched to *Proteus mirabilis*. Isolate H23WCTM6 was closely matched to *Alcaligenes faecalis*. Isolate K6BCTM4 was closely matched to *Myroides odoratimimus*. Isolates J1BCRM5 and J17WTRM6 were closely related to *Macrococcus caseolyticus*. Isolates J6WTTM5, J6BCTM5, and L10WTTM5 were closely matched to *Staphylococcus xylosus*. Isolate A9WCTM6 was closely matched to *Aeromonas veronii* (Figure 3).

### 3.3. Genetic Diversity

**Figure 4:**
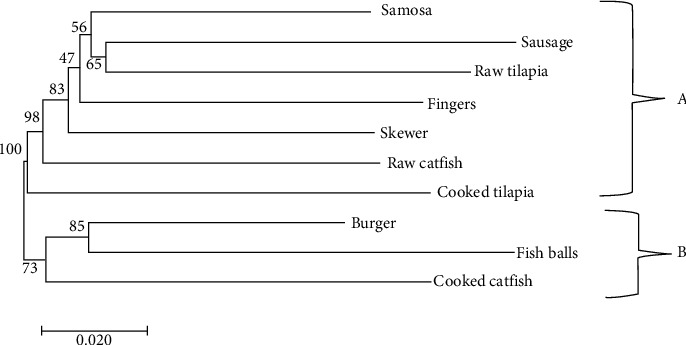
Evolutionary relationships of taxa of bacteria isolates from fish products inferred using the neighbor-joining method based on Nei unbiased genetic distance.

**Figure 5:**
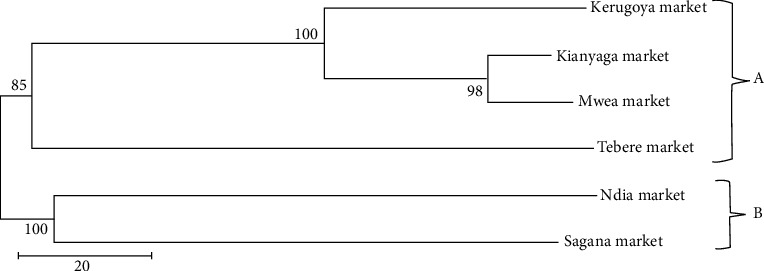
Evolutionary relationships of taxa of markets bacterial isolates inferred using the neighbor-joining method based on Nei unbiased genetic distance.

A neighbor-joining method based on Nei unbiased genetic distance using pairwise matrix clustered bacterial strains from the different fish products into two main clusters, cluster A and cluster B. Cluster A had seven bacterial isolates from different fish products. Cluster B had the least number of bacteria isolated from; burgers, fish balls, and treated catfish clustering together (Figure 4). Cluster analysis grouped bacterial isolates from Kirinyaga County into two main clusters. Bacterial strains from Kerugoya, Kianyaga market, Mwea market, and Tebere market were grouped in cluster A which had the majority. Cluster B had only two bacteria population from Ndia market and Sagana market (Figure 5).

## 4. Discussion

In this study, 158 pure bacterial isolates were obtained from fish and fish products from six markets in Kirinyaga County. A majority of the isolates were obtained from raw fish, unlike other processed fish products such as fish burgers, hotdogs, and cakes which recorded the least number of bacterial isolates. Fish products have been documented to harbor diverse bacteria, including pathogenic bacteria and other microorganisms [20]. The availability of pathogenic bacterial isolates in fish and fish products may be attributed to the environmental conditions and preservation processes used by fish handlers that promote the survival and spread of these pathogenic microbes [21]. However, it has been reported that proper fish processing methods can significantly reduce bacterial isolates present on the fish skin and other fish surfaces, hence reducing bacterial contamination [22]. Previously, Basti et al. [23] and Ghaly et al. [21] demonstrated that fish processing controls microbial growth and reduces fish contamination compared with unprocessed raw fish. In contrast, Pal et al. [24] reported the possibility of contaminating the processed fish products when processing and postprocessing are done in an unhygienic environment.

The bacteria isolated in this study had diverse morphological characteristics. Therefore, isolates with similar morphological characteristics are clustered together.

The high number of bacterial isolates from the raw fish compared to the processed fish could be due to contamination of the fish habitats. A few isolates, such as K1-WTRM5 and L12-FCCTM6, had a unique characteristic of swarming on the plate; hence, it is difficult to establish the colony size. According to Zhou et al. [25], bacteria produce extracellular substances as a defense mechanism. Based on Gram staining reaction, majority of the bacterial isolates in this study were Gram-negative. The finding agrees with those of Cabra et al. [26], where the majority of the isolated bacteria from different fish species were Gram-negative bacteria. The common genera of bacteria from the fish were those from genera *Pseudomonas*, *Flavobacterium*, and *Achromobacter* and species *Escherichia coli* and *Vibrio* sp. [27]. However, Gram-positive bacteria were also isolated in this study. According to Al-Reza et al. [28], Gram-positive bacteria in the genera *Clostridium*, *Bacillus*, and *Micrococcus* have been isolated from different fish species.

The *Bacillus* sp. were the majority of the isolates in this study. The predominance of the *Bacillus* species can be attributed to their ubiquitous nature and the ability to produce endospores which allows them to survive in fish and fish processing condition [29]. Processing of these fish poses a chance of contamination from the processing environment mainly from the fish processing handlers and processing materials. The bacteria contribute to fast food spoilage because they do not produce extracellular enzymes and toxins [1].

On the other hand, clustering of I1FcNTM1, I2SkCTM4, and L9WTM5 isolates confirms their genetic relatedness with *Bacillus amyloliquefaciens*, which is mostly isolated from the soil [30]. In the aquaculture industries, *Bacillus amyloliquefaciens* has been used in the remediation of aquaculture water [31]. The isolation of *Bacillus amyloliquefaciens* from fish products indicates a possibility of contamination. Similarly, bacterial isolates from this study clustered with organisms, *Bacillus thuringiensis* [32], *Bacillus safensis*, and *Bacillus pumilus*, confirm the diversity of isolates. The isolation of *Bacillus* sp. poses a considerable concern since these organisms have the potential of causing infections and food poisoning.

Moreover, human pathogens were also detected in these fish products. *Klebsiella* species isolated and dominant in this study was *Klebsiella oxytoca*. These organisms were isolated from samosas and sausages, suggesting possible fecal contamination during processing. Besides, the fecal contamination could be associated with poor sanitation observed from the sampled markets. This bacterial species has been cited to be the most common cause of nosocomial infections [33]. Other *Enterobacter* species and strains are known to be etiological agents of human intestinal and extra intestinal infections. Previously, these bacteria have been isolated from the tegument and gut of reared and retailed fish [34].

The isolation of *Salmonella enterica* in fish products was of health concern, and it indicates poor handling and sanitation exhibited by fish handlers. Other bacterial isolates such as F3WTRM5, M10SmCTM1, and M10SaCRM5 were closely related to *Escherichia coli*. Previously, these bacterial species have been isolated from fecal samples of humans, animals, and avian [35]. Isolation of *E. coli* in this study may indicate inadequate clean water in the markets and possible fecal contamination of fish and fish products. Also, closely related to *Proteus mirabilis* was isolated in this study. Previously, *Proteus* sp. has been isolated from urine and is known to virulence factors which enhance the adhesion of this pathogen in urinary tract thus causing the urinary tract infections (UTIs), as demonstrated by Fusco et al. [36]. *Proteus mirabilis* is a human pathogenic bacteria isolated from fish by Thongkao and Sudjaroen [37]. The pathogen has also been isolated from tilapia fish, and it could also have found its way to the fish via poor sanitation from the fish handlers.

*Aeoromonas veronii* was also isolated from the study samples. According to Tang et al. [38], the bacteria is a causative agent of bacteremia, which is a threat to human health. In addition, the bacterium has been documented as being a fish pathogen that causes hemorrhagic septicemia and epizootic ulcerative syndrome in fish, hence resulting in losses to farmers. Globally, this pathogen has been isolated from different water bodies [39]; therefore, it is in agreement with this study that *Aeromanas veronii* can be isolated from fish or fish-related products since fish are obtained from water sources which have been previously associated with this group of bacteria. Furthermore, isolate H23WCTM6 was closely related to *Alcaligenes faecalis.* According to Zhang et al. [40], this isolate has been harnessed to remove nitrogenous substances from industrial and domestic wastewater. *Alcaligenes faecalis* has also been implicated as a fish spoilage microflora as demonstrated by Austin et al. [41]; hence, it can be isolated in fish products. Studies have shown that *Staphylococcus xylosus* is among CoNS (coagulase-negative staphylococci) found in leafy vegetables such as lettuce, parsley, mint, and cress. Isolate K6BCTM4 was closely related to *Myroides odoratimimus*. It is among *Myroides* sp. predominantly isolated from different pathogen sources, including urine and wound discharges [42]. This bacterium is an important human pathogen that has been isolated from clinical specimens, and its presence in fish can be attributed to contamination due to poor handling of the fish and fish products, poor sanitation, and personal hygiene.

The majority of bacterial isolates from different fish products had high haplotype diversity. This depicts that diverse bacterial strains contaminated the fish products. Variability in genetic diversity in different fish products could be attributed to different fish products processing methods in Kirinyaga County [43]. The finding agrees with a previous study by Koo et al. [44], who reported that different beef products in South Korean retail markets had a relatively high diversity index due to differences in the fish processing methods. Other factors that contribute to the genetic diversity of the bacteria isolates from fish products include storage facilities, handling procedures after processing, packaging, and methods of transportation [40]. Poor handling and unavailability of storage facilities among the fish vendors may have contributed to this high diversity. According to Polz et al. [45], high allelic diversity may be possible within fish products with high diversity variations.

Knowing this high diversity within bacterial isolates is vital in formulating appropriate processing methods that can reduce the risks associated with bacterial contamination of fish and fish products. The limited genetic distance of bacterial isolates from fish products obtained from markets in Kirinyaga County may be attributed to the genetic recombination of some bacterial isolates. According to Polz et al. [42], horizontal gene transfer might contribute to low genetic differentiation of bacterial isolates from a common source. There was a narrow diversity of bacterial isolates from different markets within Kirinyaga County. This indicates that most of the bacterial strains isolated from fish obtained from Kirinyaga County markets were closely related and may have shared a common ancestral origin [46]. The existence of short genetic distance is also attributed to isolation of bacterial strains from a narrow host range, common geographical region, and isolation of bacteria isolates adapted to a specific common niche. However, the low variance between the bacterial strains can be advantageous in devising effective processing methods.

## 5. Conclusion

Phylogenetic analysis from the 54 bacterial morphological groups revealed the genetic diversity of the bacterial isolates from the markets in Kirinyaga County, Kenya. *Bacillus* sp. was the dominant group of bacteria. The bacterial isolates H10WTRM5 and H18 SaCTM1 were unique, and they should be subjected to complete genome sequencing to confirm their identity and investigate possible risks in fish products. Some human pathogens were also isolated, indicating health risks posed to the fish consumers in Kirinyaga County. Based on the level of contamination reported in this study, there is a need for continuous surveillance of the fish and fish products in these markets to prevent possible foodborne disease outbreaks in the future.

## Figures and Tables

**Table 1:** Morphological characteristics of bacterial isolates from fish products.

		Culture characteristics							Cell characterization	
Source	Isolate ID	Color	Shape	Size (mm)	Texture	Transparency	Margin	Elevation	Cell shape	Gram stain
*Raw fish*										
F1	FfNRM1	SW	R	3	FR	T	E	F	Rd	—
A1	FfNRM1	SW	R	2	FR	T	E	R	Rd	—
A4	WTRM5	CW	IR	2	M	T	U	F	Rd	+
D3B	WTRM2	Y	R	3	FR	T	E	F	Rd	—
F3	WTRM5	CW	R	3	FR	T	E	C	Rd	—
H18	WTRM5	CW	IR	4	M	T	E	R	Rd	+
J1	BCRM5	Y	IR	4	FR	O	E	C	Cc	+
H12	BCTM5	CW	R	3.5	M	T	E	R	Rd	+
M10	WTRM5	CW	R	3	FR	O	E	C	Rd	—
K1	WTRM5	W	SW		M	T		F	Rd	—
A6	WTRM6	W	R	1	FR	O	E	F	Rd	—
A11	WCRM6	W	R	2	FR	O	E	R	Rd	—
H26	WCRM6	CW	IR	3	FR	O	E	R	Rd	+
L3	WCTM1	W	R	3	M	T	E	F	Rd	—
D15	WTRM5	Y	R	3	M	O	E	F	Rd	—
A7,	FfNRM1	CR	SW	_	FR	TP	E	R	Rd	—
J12	FfcRM1	GY	R	1	FR	O	E	F	Rd	—
F1	FfcRM1	W	R	3.5	FR	T	E	F	Rd	—
A9	WCTM6	CW	R	3	FR	O	E	F	Rd	—
A10	WCTM6	W	R	2	M	T	E	R	Rd	—
L10	WTTM5	GY	IR	3	FR	O	E	R	Rd	—
H16	WCRM6	GY	R	2	M	T	E	C	Rd	—
C1	FCTM1	C	R	2	M	O	E	F	Rd	—
H3	FcCTM4	CW	IR	3.5	FR	O	E	R	Rd	+
G11	FfNRM1	GY	SW		FR	T			Rd	—
J17	WTRM6	Y	IR	3	FR	O	E	C	Cc	+
K1	WTRM5	Y	SW		FR	T			Rd	—
*Sausage*										
B1	SaCTM1	W	R	3	G	T	E	R	Rd	—
B2	SaCRM1	CW	R	4	FR	T	E	R	Rd	—
*Samosa*										
D1	SmCTM1	CW	R	2	FR	T	U	F	Rd	—
D2	SmCTM2	CW	R	3	M	O	U	F	Rd	—
H19	SmCTM1	CW	IR	2	M	T	E	F	Rd	+
L13	SmCTM1	Y	R	3.5	FR	T	E	C	Rd	—
L12	FcCTM6	W	SW		M	T			Rd	—
*Skewer*										
F2	SkCTM4	W	R	2	FR	T	E	F	Rd	—
H17	WTTM(5)	CW	IR	3	FR	T	U	F	Rd	+
H4	SkCTM4	W	IR	2.5	FR	T	E	F	Rd	+
I2	SkCTM4	CW	IR	4	M	T	U	R	Rd	+
*Treated (deep fried)*										
E1, E2	WCTM1	CW	R	1	G	T	E	F	Rd	—
E2	WCTM1	CR	IR	6	FR	O	U	R	Rd	—
H23	WCTM5	CR	IR	4	M	T	U	F	Rd	+
J6	WTTM5	GY	R	5	M	O	E	R	Cc	+
H5	WTTM5	CR	IR	3	FR	T	E	R	Rd	+
H13	SACTM3	W	SW		M	T		F	Rd	—
*Balls*										
H1	BcTM4	CW	IR	2	G	T	U	F	Rd	+
I1	BcTM4	CW	IR	3	FR	T	U	R	Rd	+
H7	BcTM5	CW	R	4.5	M	O	E	R	Rd	+
H6	BcTM5	CW	IR	6	M	O	E	R	Rd	+
*Fried*										
H8	WTTM5	P	R	3	FR	T	E	F	Rd	+
H10	WTTM5	CW	IR	5	M	O	E	R	Rd	—
M8	WTTM5	P	R	3.5	FR	T	U	E	Rd	+
H11	SMCTM5	CW	IR	3	FR	O	U	R	Rd	+
H15	WWTM5	CW	IR	4.5	M	O	E	R	Rd	—
M9	SACRM5	P	R	1	G	T	E	C	Rd	+
G7	SMCRM5	GY	R	3	FR	O	E	C	Rd	—
D16	WTTM5	Y	IR	5	M	T	E	F	Rd	—
D15	WTRM5	Y	R	3	FR	T	E	F	Rd	—
*Cakes*										
C1	FcCTM1	C	IR	3	FR	T	U	F	Rd	—
H14	SmCRM5	CW	IR	4	M	T	U	F	Rd	+
*Burger*										
H2	BuCTM4	CW	IR	5	M	O	E	R	Rd	+
*Hot dog*										
G1	HdCTM4	Y	R	3	FR	O	E	R	Rd	—
*Soup*										
C3	SMCTM1	SW	R	3	FR	T	E	F	Rd	—
K3	SMCTM4	CW	IR	5	M	O	U	R	Rd	+
M7	SMCTM3	P	R	3	G	O	E	R	Rd	—

Key: SW: slightly whitish; CW: cream white; GY: golden yellow; W: white; Y: yellow; P: pink; SW: swarming; R: round; IR: irregular; FM: firm; M: mucoid; O: opaque; T: translucent; TP: transparent; E: entire; U: undulate; F: flat; R: raised; C: convex; Rd: rods; Cc: cocci; −: Gram-negative; +: Gram-positive.

**Table 2:** Biochemical and Gram staining characteristics of the bacterial isolates.

Tests		TSI				SIM			MIU					MR-VP		Possible identity	
Isolates	Origin	S	B	H_2_S	G	H_2_S	I	M	M	I	U	U	C	MR	VP	Cell shape	
A4	WTRM5	Y	Y	+	+	+	—	+	+	—	+	+	+	+	—	Rd	*Proteus* sp.
A6	WTRM6	P	Y	+	+	+	—	+	+	—	+	—	+	+	—	Rd	*Citrobacter* sp.
A7	FtNRM1	Y	Y	+	—	+	—	+	+	—	+	+	—	+	—	Rd	*Proteus* sp.
A9	WCTM6	Y	Y	+	—	+	+	+	+	+	+	+	+	+	—	Rd	*Aeromonas* sp.
A11	WCRM6	P	Y	+	+	+	+	+	+	+	+	+	—	—	+	Rd	*Citrobacter* sp.
B1	SaCTM1	P	Y	+	+	+	—	+	+	—	—	—	—	+	—	Rd	*Citrobacter* sp.
B2	SmCTM1	Y	Y	—	—	—	+	—	—	+	+	+	+	—	+	Rd	*Klebsiella* sp.
C1	FCTM1	P	Y	+	+	+	—	+	+	—	—	—	—	+	—	Rd	*Salmonella* sp.
C2	FCTM5	P	Y	+	+	+	+	+	+	+	+	+	+	+	—	Rd	*Citrobacter* sp.
C3	SmCTM1	P	Y	+	+	+	—	+	+	—	+	+	—	+	—	Rd	*Citrobacter* sp.
D1	SmCTM1	Y	Y	—	+	—	—	+	+	—	—	—	—	—	+	Rd	*Citrobacter* sp.
D2	SmCTM2	Y	Y	—	+	—	—	+	+	—	—	—	—	—	+	Rd	*Enterobacter* sp.
D3B	WTRM2	Y	Y	—	+	—	—	+	+	—	—	—	—	—	+	Rd	*Enterobacter* sp.
E1	WCTM1	Y	Y	+	+	+	—	+	+	—	—	—	+	—	+	Rd	*Bacillus* sp.
E2	WCTM1	P	Y	—	+	+	—	+	+	—	—	—	—	+	—	Rd	*Enterobacter* sp.
F1	FfNRM1	P	Y	—	—	—	+	—	—	+	—	—	—	—	—	Rd	*Citrobacter* sp.
F2	SkCTM4	P	Y	—	+	—	+	—	—	+	—	—	—	+	—	Rd	*Citrobacter* sp.
F3	WTRM5	Y	Y	—	+	—	—	—	—	—	—	—	—	+	—	Rd	*Escherichia* sp.
G7	SmCRM5	Y	Y	+	+	+	—	+	+	+	+	+	—	+	—	Rd	*Raoultella* sp.
G11	FfNRM1	Y	Y	+	+	+	—	+	+	—	+	+	—	+	—	Rd	*Proetus* sp.
H2	BuCTM4	Y	Y	+	+	+	—	+	+	—	—	+	+	—	+	Rd	*Bacillus* sp.
H3	FcCTM4	Y	Y	+	+	+	—	+	+	—	—	+	+	—	+	Rd	*Bacillus* sp.
H14	SmCRM5	Y	Y	—	+	—	—	—	—	—	—	+	—	—	—	Rd	*Bacillus* sp.
H15	WTTM5	P	Y	—	+	—	—	+	+	+	+	+	+	+	+	Rd	*Bacillus* sp.
H16	WCRM6	P	Y	+	+	+	+	+	+	+	+	+	—	—	+	Rd	*Bacillus* sp.
H17	WTTM5	Y	Y	—	—	—	—	—	—	—	—	—	+	—	—	Rd	*Bacillus* sp.
H18	WTRM5	P	Y	—	—	—	—	—	—	+	—	+	+	+	—	Rd	*Klebsiella* sp.
H19	SmCTM1	Y	Y	—	+	—	—	—	—	—	—	+	—	—	—	Rd	*Bacillus* sp.
H23	WCTTM6	P	Y	—	—	—	—	—	—	+	+	+	+	+	—	Rd	*Alcaligenes* sp.
H26	WCRM6	P	Y	+	—	+	+	+	+	+	+	+	—	+	—	Rd	*Bacillus* sp.
I2	SKCTM4	Y	Y	—	+	—	—	—	—	—	+	+	—	—	—	Rd	*Bacillus* sp.
J1	BCRM5	Y	Y	+	—	+	+	+	+	+	+	+	—	—	+	Cc	*Macrococcus* sp.
J6	WTTM 5	Y	Y	—	+	—	—	—	—	—	+	+	—	—	—	Cc	*Staphylococcus* sp.
J12	FFCRM6	Y	Y	—	+	—	+	—	+	—	—	—	—	+	—	Rd	*Bacillus* sp.
J17	WTRM6	P	Y	—	+	—	+	+	+	+	+	+	—	+	—	Rd	*Bacillus* sp.
K1	WTRM5	P	Y	—	+	—	+	+	—	+	—	—	—	+	—	Rd	*Bacillus* sp.
K3	SMCTM4	Y	Y	+	+	+	+	—	—	—	—	+	—	—	+	Rd	*Bacillus* sp.
L3	WCTM 4	Y	Y	—	+	—	—	+	+	—	—	—	—	—	+	Rd	*Enterobacter* sp.
L10	WTTM 5	Y	Y	—	+	—	—	—	—	—	+	+	—	—	—	Cocci	*Staphylococcus* sp.
L13	SMCTM1	Y	Y	—	+	—	—	+	+	—	—	—	—	—	+	Rd	*Enterobacter* sp.
M7	SmCTM5	Y	Y	—	+	—	—	—	—	—	—	+	—	—	—	Rd	*Klebsiella* sp.
M8	WTTM5	P	Y	+	+	+	+	+	+	+	+	+	—	—	+	Rd	*Klebsiella* sp.
M9	SACRM 5	P	Y	—	+	—	+	+	+	+	—	—	—	—	+	Rd	*Bacillus* sp.
M10	WTRM5	P	Y	+	+	+	+	+	+	+	+	+	+	—	+	Rd	*Escherichia* sp.

Key: P: pink; Y: yellow; TSI: triple sugar iron; S: slant; B: butt; H2S: hydrogen sulfide; G: gas; SIM: sulfide indole motility; I: indole; M: motility; MIU: motility indole urease; U: urease; C: citrate; MR: methyl red; VP: Vogues Proskauer; +: positive; −: negative.

## Data Availability

The data used to support the findings of this study are available from the corresponding author upon request.
